# Development and Analytical Evaluation of a Novel Droplet Digital PCR Assay for Molecular Detection of *Rickettsia* spp. in Dogs and Cats from the Canary Islands

**DOI:** 10.3390/vetsci13080761

**Published:** 2026-07-30

**Authors:** Alma García-Ramos, Omar García-Pérez, Angélica Domínguez-de-Barros, Malena Gajate-Arenas, Candela Sirvent-Blanco, José E. Piñero, Jacob Lorenzo-Morales, Elizabeth Córdoba-Lanús

**Affiliations:** 1 Instituto Universitario de Enfermedades Tropicales y Salud Pública de Canarias, Universidad de La Laguna, 38029 San Cristóbal de La Laguna, Spain; agarcira@ull.edu.es (A.G.-R.); ogarciap@ull.edu.es (O.G.-P.); adomingb@ull.edu.es (A.D.-d.-B.); ogajatea@ull.edu.es (M.G.-A.); alu0101234656@ull.edu.es (C.S.-B.); jpinero@ull.edu.es (J.E.P.); jmlorenz@ull.edu.es (J.L.-M.); 2 Centro de Investigación Biomédica en Red de Enfermedades Infecciosas (CIBERINFEC), Instituto de Salud Carlos III, 28029 Madrid, Spain; 3 Departamento de Obstetricia y Ginecología, Pediatría, Medicina Preventiva y Salud Pública, Toxicología, Medicina Legal y Forense y Parasitología, Facultad de Ciencias de la Salud, Universidad de La Laguna, 38200 San Cristóbal de La Laguna, Spain; 4 Departamento de Bioquímica, Microbiología, Biología Celular y Genética, Facultad de Ciencias, Universidad de La Laguna, 38200 San Cristóbal de La Laguna, Spain

**Keywords:** *Rickettsia*, rickettsiosis, ddPCR, One Health, emerging diseases

## Abstract

*Rickettsia* is an obligate intracellular genus of bacteria responsible for rickettsioses, a group of emerging vector-borne diseases. Their diagnosis remains challenging because of non-specific clinical manifestations, serological cross-reactivity, and low bacterial loads in clinical samples. In this study, a novel droplet digital polymerase chain reaction (ddPCR) assay was developed for the molecular detection of *Rickettsia* spp., and its performance was evaluated using clinical blood samples collected from dogs and cats in the Canary Islands, Spain. Primers and probes targeting the 16S ribosomal RNA (rRNA) were designed and optimized for ddPCR amplification. Analytical performance was assessed by optimising primer and probe concentrations, annealing temperature, specificity, and the limit of detection (LOD). Comparative analysis between quantitative PCR (qPCR) and ddPCR was also performed. The optimized assay employed primer and probe concentrations of 900 nM and 250 nM, respectively, with an annealing temperature of 56 °C. The assay achieved an analytical detection limit of 0.064 ng of clinical sample DNA, although sporadic positive droplets were still detectable at lower concentrations. Strong inverse correlations were observed between qPCR cycle threshold (C_T_) values and ddPCR absolute quantification for both plasmid DNA (r = −0.997) and clinical sample DNA (r = −0.983). A total of 170 blood samples collected from domestic animals in the Canary Islands, with clinical suspicion of rickettsiosis, were analysed. Six canine samples (3.5%) tested positive for *Rickettsia* spp. The ddPCR assay developed in this study showed promising analytical performance for the molecular detection of *Rickettsia* spp. DNA and has the potential to become a valuable molecular tool for veterinary diagnostics and epidemiological investigations within a One Health framework. Further analytical and clinical validation studies are required before its implementation as a routine diagnostic assay.

## Simple Summary

Rickettsioses are emerging vector-borne diseases affecting both animals and humans. They are caused by bacteria species of *Rickettsia*, a genus which is mainly transmitted by ticks, fleas, and other arthropods. Diagnosing these infections can be difficult because the clinical signs are often non-specific and only small amounts of bacterial DNA may be present in blood samples. In this study, we developed a new laboratory diagnostic method based on droplet digital PCR (ddPCR), a highly sensitive molecular technique designed to detect very low levels of *Rickettsia* DNA. The assay was applied to blood samples from 170 dogs and cats in the Canary Islands (Spain) with suspected rickettsial infection, and *Rickettsia* spp. DNA was detected in six dogs. Although further validation is required before routine diagnostic use, this method shows promise as a tool for veterinary diagnostics and could support future surveillance of *Rickettsia* infections in animals, contributing to a One Health approach that recognizes the close links between animal and human health.

## 1. Introduction

Rickettsioses are a group of emerging diseases caused by Gram-negative obligate intracellular bacteria in the genus *Rickettsia*. These bacteria are primarily transmitted through the bite of hematophagous arthropods that act as vectors, which become infected during blood feeding. There is a wide range of vectors associated with *Rickettsia* spp., such as lice, fleas, ticks or mites [1,2]. Moreover, different mammalian hosts, such as rodents, dogs, livestock, wild mammals, and birds, may act as reservoirs depending on the specific *Rickettsia* species involved [3]. Importantly, humans may become accidental hosts, being infected through contact with the saliva or faeces of infected vectors and developing diseases of varying severity [4].

Rickettsial diseases are difficult to quantify globally due to their imprecise diagnosis, nonspecific manifestations, and geographical variability. Furthermore, freight transport, climate change, globalization, and environmental change facilitate the colonization of new areas by vectors and reservoirs, as well as by *Rickettsia* spp. In Asia, for example, rickettsial infections are the second most common cause of non-malarial diseases, after dengue [5]. In addition, in the United States of America (USA), Rocky Mountain spotted fever (RMSF; *Rickettsia rickettsii*) is the most severe and common form, with an incidence reaching approximately 7 cases per million inhabitants per year between 2000 and 2007 [6] and a potentially high case fatality of 20–25% if left untreated [7].

Rickettsiosis courses with typical signs and symptoms of tick-borne diseases, including fever, muscle pain or headache. In addition, it is common in patients with *Rickettsia* spp. infection to have different types of rashes, including macular, maculopapular, and petechial rash. Even certain species of the genus *Rickettsia* can produce a necrotic lesion denominated the crust [1,2,3]. Depending on the clinical manifestations, the pathogenic bacteria of the genus *Rickettsia* can be categorized into three distinct groups. The first one is the typhus group, which consists of *R. prowazekii* and *R. typhi*, responsible for classic typhus. Second, the spotted fever group (SFG), which is composed of more than 30 species, including *R. rickettsii* as the etiological agent of RMSF, *Rickettsia conorii* or *Rickettsia parkeri*. Finally, the transitional group is composed of *Rickettsia akari*, *Rickettsia felis* or *Rickettsia australis* [8].

The Canary Islands constitute a subtropical area and are particularly prone to colonization by different types of vectors due to climate change, tourism, and the transport of goods [1,3,9]. These factors represent the first step in parasite dissemination, given that the Canary Islands are clearly at risk. *Rickettsia* spp. are pathogens whose prevalence could increase under these conditions and play a crucial role in public health due to its zoonotic potential [2,3,4].

The study of *Rickettsia* spp. in domestic animals represents a critical component of public health, as canids and felids may act as reservoirs of these pathogens, and they come into close and frequent contact with humans [10]. Likewise, the genus *Rickettsia* plays a significant epidemiological role, given its impact on domestic dogs and their epizootic and zoonotic potential [11]. Therefore, and in line with the One Health framework, accurate diagnostic methods and appropriate treatment of infectious diseases in domestic animals become highly relevant to prevent the spread of these bacteria among both humans and animals [12].

As previously mentioned, the diagnosis of rickettsiosis remains challenging. The signs and symptoms of these illnesses are typical of vector-borne diseases and lack specific clinical features, thereby complicating diagnosis. To date, serology has been the gold standard for the diagnosis of rickettsiosis. However, serological methods that detect antibodies against *Rickettsia* spp. present certain disadvantages, such as a window interval between infection and the development of detectable antibody levels, cross-reactivity among species, variable sensitivity and specificity depending on the method (Immunofluorescence assay (IFAT), Enzyme-linked immunosorbent assay (ELISA), or commercial kits), persistent antibodies from past infection, or difficulty of interpretation in endemic areas due to high seroprevalence [3,13,14,15].

On the other hand, molecular diagnostic methods offer numerous advantages over serological techniques currently used to detect these microorganisms, particularly their high sensitivity and specificity [13]. In this way, droplet digital polymerase chain reaction (ddPCR) provides several benefits over other PCR methods, including absolute quantification, superior sensitivity, and high analytical precision. In addition, its reproducibility and robustness facilitate more reliable comparisons between samples and across experiments [16,17]. Overall, ddPCR is a powerful tool for improving the molecular detection of critical bacteria and protozoa, as previously reported for similar agents, including *Ehrlichia canis* [18], *Bartonella* spp. [19,20], *Borrelia* spp. and *Babesia* spp. [20], and *Wolbachia* spp. [21]. The high analytical sensitivity and increased tolerance to PCR inhibitors of ddPCR may enhance the detection of *Rickettsia* spp. in animal blood samples, particularly important given the low bacterial DNA concentrations typically found during the transient phase of rickettsial bacteraemia.

Given the above, this study aimed to firstly develop a ddPCR assay for the rapid and effective detection of *Rickettsia* spp. in domestic animals. Secondly, the assay was applied to clinical samples from canids and felids collected in the Canary Islands (Spain) to validate its performance under field conditions and to confirm the molecular detection of the pathogen in naturally infected animals.

## 2. Material and Methods

### 2.1. Primer and Probe Design

Primers and the hydrolysis probe were designed using the Primer3Plus program (https://www.primer3plus.com/, accessed on 10 October 2024) after multiple sequence alignment of representative *Rickettsia* 16S ribosomal RNA (rRNA) gene sequences retrieved from GenBank. Conserved regions shared among *Rickettsia* spp. were selected as candidate targets. The specificity of the selected oligonucleotides was subsequently assessed in silico using BLASTn (https://blast.ncbi.nlm.nih.gov/Blast.cgi?PROGRAM=blastn&BLAST_SPEC=GeoBlast&PAGE_TYPE=BlastSearch, accessed on 10 October 2024) against the NCBI nucleotide database to confirm broad coverage of *Rickettsia* spp. while minimizing potential cross-reactivity with non-target bacterial sequences. Because the target region is highly conserved, the assay was intentionally designed for genus-level detection rather than species differentiation. The primers and probe used in this study were: forward 5′-ATTTGCCAGCGGATAATGCC-3′, reverse 5′-CGAGTTGCAGAGAACAATCCG-3′ and probe 5′-FAM-TGTTTACAGAGGGAAGCAAGACGGC-BHQ1-3′. The expected size of the amplicon was 201 base pairs.

### 2.2. Sample Collection

**Figure 1:**
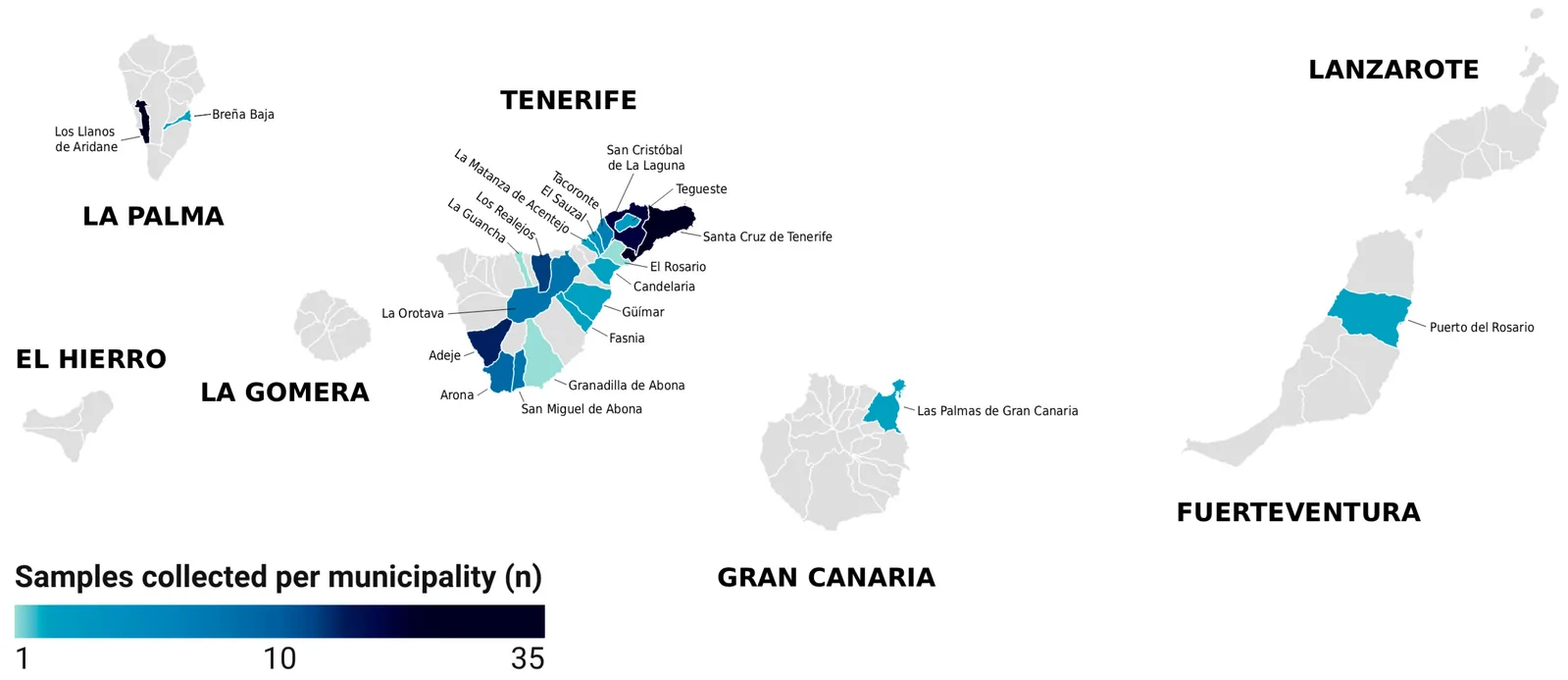
Geographical distribution of collected samples in the Canary Islands, Spain. Source: CNIG [22]. Map created with Datawrapper online tool [23].

Blood samples were collected from 170 domestic animals (156 dogs and 14 cats) presenting to collaborating veterinary clinics with a clinical suspicion of rickettsiosis. Clinical suspicion was established by the attending veterinarian based on compatible clinical signs, including myalgia, anorexia (or appetite loss), fever, and/or lethargy, together with a history of tick exposure or the presence of attached ticks. As part of the routine diagnostic investigation, some animals were additionally screened for other common canine vector-borne pathogens. Whenever these tests were performed, only animals with negative results for *Anaplasma platys*, *Ehrlichia* spp., *Anaplasma* spp., *Borrelia burgdorferi*, and *Dirofilaria immitis* were included in this study. Animals were evaluated at veterinary clinics between November 2023 and July 2025 in Fuerteventura, Gran Canaria, La Palma and Tenerife islands (Spain) (Figure 1), and samples were submitted to the reference laboratory for molecular diagnosis.

Blood samples were stored at <4 °C until nucleic acid isolation with the NucleoSpin Blood Columns kit (Macherey-Nagel, Düren, Germany). DNA samples were stored at −20 °C until molecular diagnosis was performed.

### 2.3. Ethical Statement

Biological samples have been taken by qualified veterinary personnel as part of routine veterinary practice, and animals were treated appropriately according to the Spanish legislation and the EU Directive (2010/63/UE) on “Protection of Animals Used for Experimental and Other Scientific Purposes”. The study complies with the ARRIVE (Animal Research: Reporting of In Vivo Experiments) guidelines [24] developed by the NC3Rs (National Centre for the Replacement and Refinement and Reduction of Animal Research) [25]. Data samples were anonymized to ensure the objectivity of the subsequent blind molecular assay.

### 2.4. Conventional PCR

Primer efficiency was validated using conventional PCR (cPCR), which was used exclusively for assay optimization. Reactions were set up in a total volume of 25 μL, containing 1X Kapa Taq Buffer A with Mg (10X) (Kapa Biosystems Inc., Wilmington, MA, USA), 0.12 μM primer forward, 0.12 μM primer reverse, 0.1 mM dNTP, 2 μL DNA template, 0.5 units Kapa Taq DNA Polymerase (Kapa Biosystems Inc., Wilmington, MA, USA), and PCR-grade water up to 25 μL.

The PCR was performed in a ProFlex PCR System thermocycler (ThermoFisher Scientific Inc., Waltham, MA, USA) under the following conditions: 1 cycle of 5 min. at 94 °C, followed by 35 cycles of 30 s. at 94 °C, 30 s. at 60 °C and 30 s. at 72 °C, 1 cycle of 5 min. at 72 °C and infinite hold at 4 °C. The PCR products were analysed by electrophoresis on a 1% agarose gel stained with GelRed (Biotium Inc., Fremont, CA, USA) and visualized under UV light with ChemiDoc^TM^ MP Imaging System (Bio-Rad Laboratories, Hercules, CA, USA). peqGOLD 100 bp DNA Ladder (VWR, Radnor, PA, USA) was used to verify the size of the DNA amplicon.

### 2.5. Positive Control

As a positive control, we used a positive clinical sample for *Rickettsia conorii* (kindly provided by the National Microbiology Centre, ISCIII, 28222 Madrid, Spain [26]). This amplicon was cloned into a pGEM^®^-T Vector System (Promega Corporation, Madison, WI, USA). First, the PCR product was purified from agarose gel with GFX^TM^ PCR DNA and Gel Band Purification Kit (Illustra, GE Healthcare Bio-Sciences, Pittsburgh, PA, USA) following the manufacturer’s instructions. Secondly, ligation with pGEM^®^-T Vector and transformation of JM109 High Efficiency Competent Cells, provided by the pGEM^®^-T Vector System (Promega Corporation, Madison, WI, USA), were performed according to the manufacturer’s instructions. Subsequently, plasmid DNA was extracted from JM109 cells using the NucleoSpin^®^ Plasmid kit (Macherey-Nagel, Düren, Germany) and its concentration was measured with a NanoDrop Lite Spectrophotometer (ThermoFisher Scientific Inc., Waltham, MA, USA). Finally, plasmid DNA was paired-end sequenced using specific primers on a SeqStudio Genetic Analyzer (ThermoFisher Scientific Inc., Waltham, MA, USA) to confirm the success of the process. Resulting DNA sequences were blasted against the NCBI database.

To determine the optimal concentration for the positive control, a ten-fold serial dilution was prepared ranging from 0.1 to 0.1 × 10^−7^ ng/μL, and 2 μL of each dilution were used for ddPCR. Moreover, the copies/μL of 16S rRNA sequence in these dilutions were calculated using the formula previously described [27]:

$$Copies/mL=Concentration\;of\;Plasmid\;(g/mL)\times \frac{Avogadro’s\;Constant}{Molecular\;Weight\;of\;Plasmid\;(\frac{g}{mol})}$$

The Sequence Manipulation Suite (https://www.bioinformatics.org/sms2/dna_mw.html, accessed on 1 December 2025) was used to estimate the molecular weight of double-stranded 16S rRNA gene amplicons, yielding 124,200.35 g/mol.

### 2.6. Real-Time PCR

Real-time PCR was performed using the QuantStudio™ 3 Real-Time PCR System (Thermo Fisher Scientific, Waltham, MA, USA). Each sample was analyzed in duplicate in a reaction mixture containing 2 μL of DNA extracted from the clinical sample, 1X SsoAdvanced™ Universal Probes Supermix (2X) (Bio-Rad Laboratories, Hercules, CA, USA), 400 nM of each primer, 125 nM of probe, and PCR-grade water to a final volume of 10 μL. Amplification was carried out under the following cycling conditions: an initial denaturation step at 95 °C for 3 min, followed by 40 cycles of 95 °C for 10 s and 60 °C for 60 s, with fluorescence acquisition performed during the annealing/extension step. Two relative standard curves were generated under the same conditions using a 10-fold serial dilution of plasmid DNA (2 × 10^−1^ to 2 × 10^−7^ ng) and a 5-fold serial dilution of a positive clinical sample (40 to 0.032 ng).

### 2.7. Droplet Digital PCR Optimization

Droplet digital PCR optimisation was performed using the Bio-Rad QX600 Droplet Reader (Bio-Rad Laboratories, Hercules, CA, USA). Optimal conditions for the amplification of the 16S DNA fragment of *Rickettsia* spp. were established by performing tests with primer and probe concentration gradients, an annealing temperature gradient, and subsequently determining the limit of detection (LOD) for clinical sample DNA. For reliable threshold determination, clear separation between the positive and negative droplet populations was required. Based on this criterion, the threshold was set at 578. For the detection of *Rickettsia* spp. DNA, the Direct Quantification (DQ) method was applied. Analyses were considered valid when approximately 17,000–20,000 accepted droplets were obtained.

### 2.8. Primer and Probe Concentration Gradient

Each 22 μL reaction contained 20 ng of DNA extracted from a positive clinical sample, 1X ddPCR Supermix for Probes (No dUTP) (Bio-Rad Laboratories, Hercules, CA, USA), 200, 450 and 900 nM of each primer, 125 and 250 nM of probe, and PCR-grade water to reach the final volume. The ddPCR mix was thoroughly mixed and centrifuged for 3 min at 2500 rpm. Subsequently, the 20 μL of the reaction mix was loaded into the sample wells of the cartridge, and 70 μL of Droplet Generation Oil for Probes (Bio-Rad Laboratories, Hercules, CA, USA) was added to each oil well. Droplets were generated using a QX200 Droplet Generator (Bio-Rad), yielding 40 μL of droplet emulsion, which was transferred to a 96-well plate and sealed with a PX1 PCR Plate Sealer (Bio-Rad Laboratories, Hercules, CA, USA). PCR cycling was performed in the PTC Tempo Deepwell Thermal Cycler (Bio-Rad Laboratories, Hercules, CA, USA), and fluorescence was read in the FAM channel using the Bio-Rad QX600 Droplet Reader (Bio-Rad Laboratories, Hercules, CA, USA).

### 2.9. Annealing Temperature Gradient

Using the optimal primer and probe concentrations defined above, an annealing temperature gradient from 55 to 63 °C was applied. Reactions contained 20 ng of DNA from a positive clinical sample, and all steps followed those described above.

### 2.10. Specificity Evaluation

To further validate the specificity of the primers and probe design, a ddPCR assay was performed using DNA extracted from positive *Anaplasma* spp. and *Ehrlichia* spp. samples under the previously established optimal conditions.

### 2.11. Plasmid DNA Positive Control

To determine the most appropriate concentration of the plasmid DNA control for routine ddPCR, a ten-fold serial dilution series (2 × 10^−1^ to 2 × 10^−7^ ng) was evaluated.

### 2.12. Limit of Detection for Clinical Samples

Finally, the limit of detection for clinical sample DNA was evaluated under the optimized primer/probe concentrations and annealing temperature. A five-fold serial dilution ranged from 40 to 2.6 × 10^−3^ ng was performed, and 2 μL of each dilution was used for the ddPCR assay.

### 2.13. Statistical Analysis

A linear regression analysis was performed to assess the correlation between cycle threshold (C_T_) values obtained by qPCR and the absolute target concentrations (copies/µL) quantified by ddPCR. Serial dilutions, covering the dynamic range of the assay, were analyzed by both qPCR and ddPCR, using the same target-specific primers and probe set, as well as the same plasmid DNA for assay optimization.

Absolute target concentrations obtained by ddPCR (expressed as copies/µL) were log_2_-transformed before analysis to ensure linearity with C_T_ values. Linear regression analysis was conducted using C_T_ values as the dependent variable and log_2_-transformed ddPCR copy numbers as the independent variable. The strength of the association between variables was assessed using the Pearson correlation coefficient (r) and the coefficient of determination (R^2^).

The Bland–Altman method was used to compare ddPCR (copies/µL) and qPCR (C_T_ values) results. This comparison test was used in this study to represent the difference between the log_2_ (copies/µL) obtained by ddPCR and the qPCR cycle threshold values, plotted against the mean of these two values. Technical reproducibility of ddPCR and qPCR was evaluated using duplicate measurements of a serial DNA dilution, and intra-assay variability was expressed as the coefficient of variation (CV).

## 3. Results

### 3.1. In Silico Design and Experimental Validation of Primers and Probes

Based on an extensive review of the literature, the 16S rRNA sequence was selected as a target for the specific detection of the genus *Rickettsia*, as several rickettsial species share more than 98.1% sequence identity [28,29].

Primer efficiency was assessed using conventional PCR. The electrophoresis of the amplification products revealed a single amplicon of the expected size, confirming the specificity of the primer set. To further validate the identity of the amplified fragment, the amplified plasmid DNA sequences were analysed using BLAST (https://blast.ncbi.nlm.nih.gov/Blast.cgi, accessed on 9 March 2025) against the NCBI nucleotide database. The alignment results demonstrated 95–98% identity with several *Rickettsia* spp.

### 3.2. Real-Time PCR

**Figure 2:**
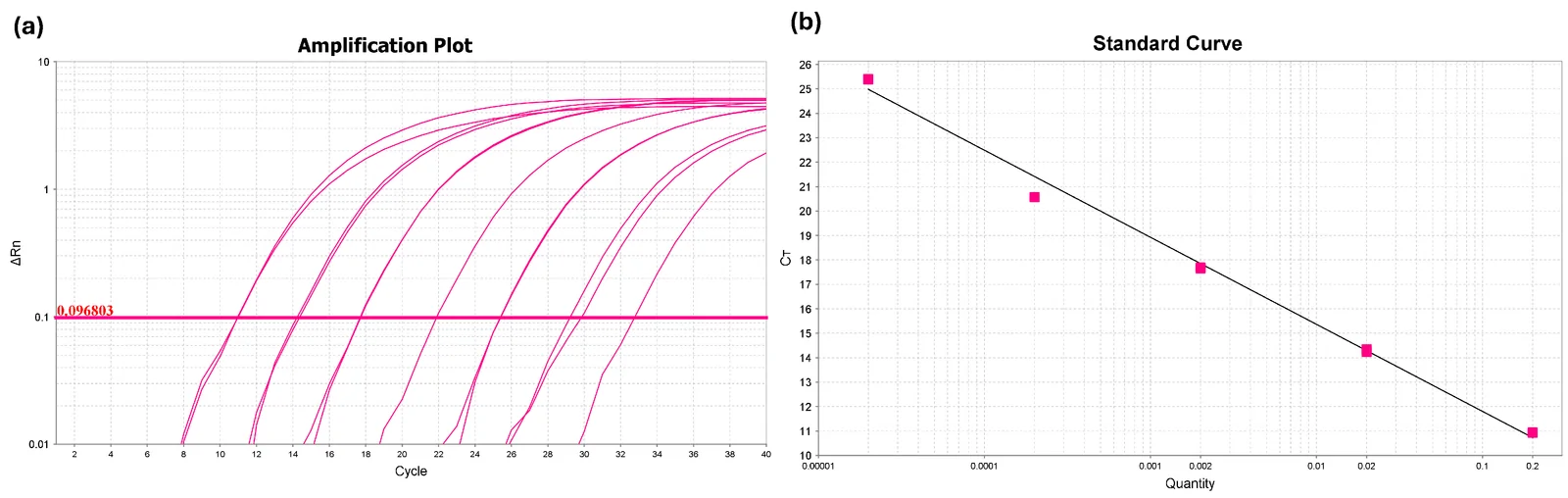
Representative qPCR standard curve generated from serial dilutions of *Rickettsia* spp. plasmid DNA (0.2 to 0.2 × 10^−7^ ng). (**a**) Amplification plot. (**b**) Standard curve (slope of −3.563; R^2^ = 0.995, E = 90.802%).

The standard curve obtained from the amplification of *Rickettsia* spp. plasmid DNA is shown in Figure 2. The plasmid DNA standard curve shows a slope of −3.563 and a correlation coefficient (R^2^) of 0.995, demonstrating high amplification efficiency (E) (90.802%) and linearity. On the other hand, the resulting standard curve from a positive sample shows a slope of −3.412, a correlation coefficient (R^2^) of 0.937 and an efficiency (E) of 96.354%. The limit of detection of qPCR for positive clinical samples was 0.064 ng of DNA, and samples with C_T_ values <36 were considered positive.

### 3.3. Development and Optimization of a ddPCR Assay

#### 3.3.1. Primer and Probe, and Annealing Temperature Gradient

**Figure 3:**
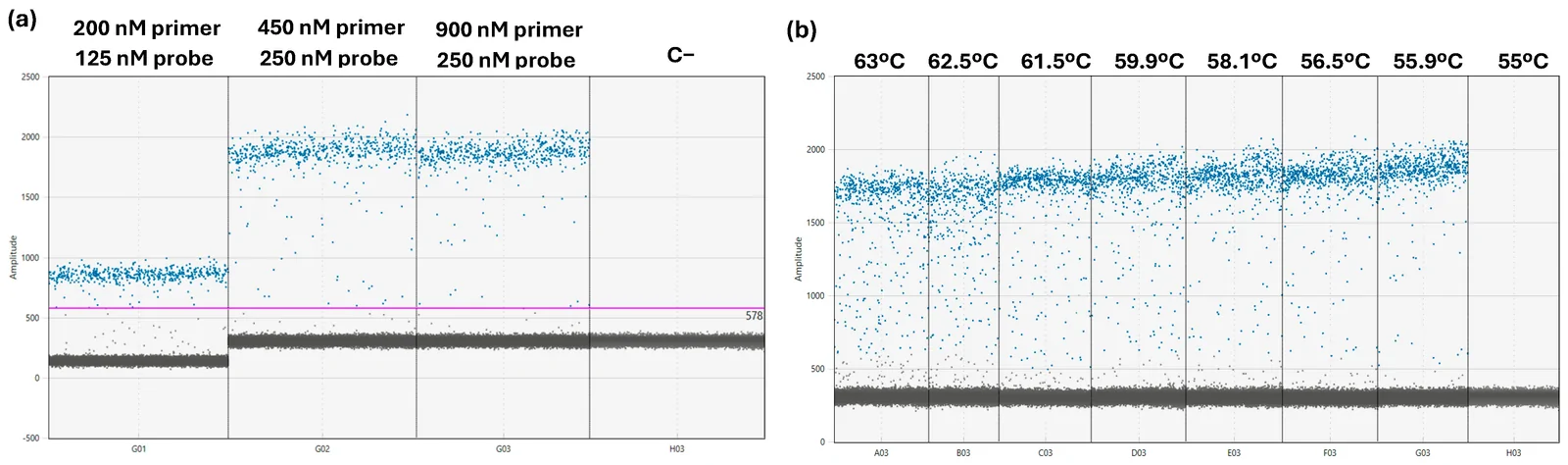
Droplet digital PCR Optimization. (**a**) Representative ddPCR plots illustrating the distribution of positive (blue) and negative (black) droplets obtained using different primer and probe concentrations: (G01) 200 nM primer, 125 nM probe, (G02) 450 nM primer, 250 nM probe, and (G03) 900 nM primer, 250 nM probe. The final well served as the negative control (C−). (**b**) ddPCR droplet distributions used for annealing temperature optimization between 55 and 63 °C. All reactions contained 10 ng of DNA, and the assay performed at 55 °C served as the negative control.

To achieve optimal amplification performance during ddPCR, several parameters were systematically optimised, including primer and probe concentrations as well as the annealing temperature. Optimisation was carried out using a known DNA sample positive for *Rickettsia conorii*, enabling a controlled evaluation of assay performance. Among the tested conditions, primer and probe concentrations of 900 nM and 250 nM, and an annealing temperature of 56 °C provided the most efficient amplification, as observed by a clear separation between positive and negative droplets, yielding the highest number of positive droplets when compared with the other conditions evaluated (Figure 3). Furthermore, an annealing temperature of 56 °C exhibited a significant reduction in rain, defined as intermediate droplets that typically arise under suboptimal amplification conditions.

#### 3.3.2. Specificity Evaluation

We confirmed that DNA extracted from *Anaplasma* spp. and *Ehrlichia* spp., bacterial genera in the same order as *Rickettsia* spp., tested negative in the ddPCR assay using primers and probe designed for the specific detection of the genus *Rickettsia*.

#### 3.3.3. Limit of Detection and Assessment of Clinical Samples

**Figure 4:**
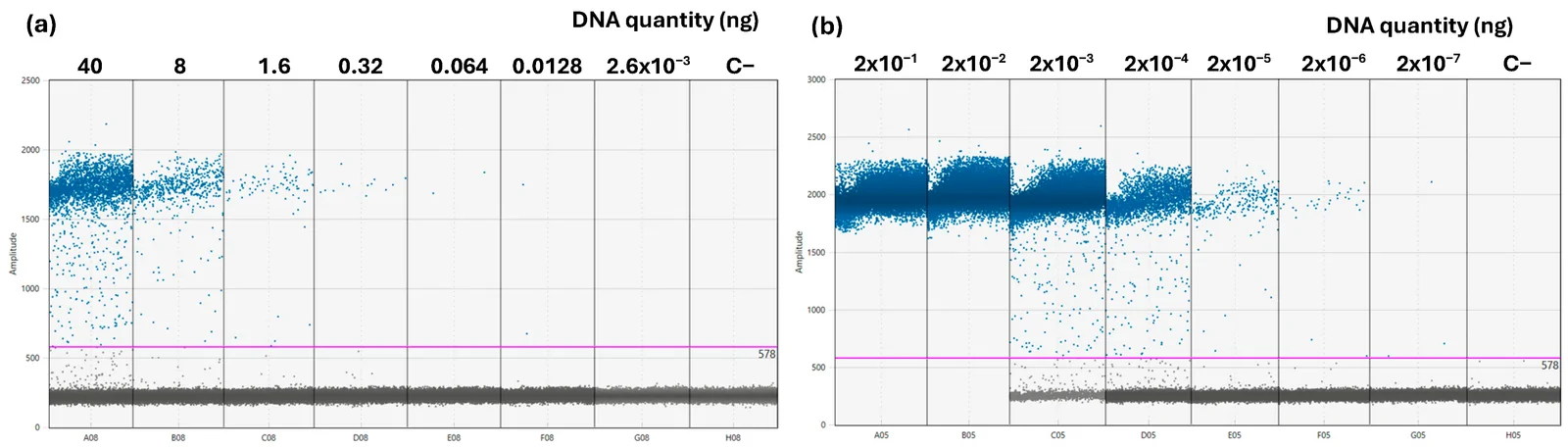
Droplet digital PCR Limit of detection, representative ddPCR plots illustrating the distribution of positive (blue) and negative (black) droplets. (**a**) ddPCR amplification of a five-fold serial dilution series ranging from 40 to 2.6 × 10^−3^ ng, with the final well serving as the negative control (C−). (**b**) ddPCR assay was performed to determine the optimal plasmid DNA concentration for routine analysis. Plasmid DNA concentrations ranged from 0.2 to 2 × 10^−7^ ng/µL, with the final well serving as the negative control (C−).

Additionally, the limit of detection (LOD) for the assay in clinical samples was determined and is presented in Figure 4a and Supplementary Table S1. This limit was set at 0.064 ng, corresponding to the lowest detectable concentration of *Rickettsia* spp. DNA that could be consistently detected across replicates. Nevertheless, positive droplets can be detected below this limit.

To evaluate clinical samples obtained from animals suspected of rickettsiosis, a plasmid DNA containing the target region was used as a positive control throughout the experiments. A ten-fold serial dilution series was evaluated to determine the most appropriate concentration for this control for routine ddPCR use. Based on the droplet profiles and quantification stability across dilutions, the optimal positive control concentration was determined to be 2 × 10^−5^ ng of plasmid DNA, corresponding to 4.85 × 10^4^ copies/μL (Figure 4b). A clear, reproducible positive signal, without droplet oversaturation, was observed.

### 3.4. Comparative Statistical Analysis Between Both Techniques

**Figure 5:**
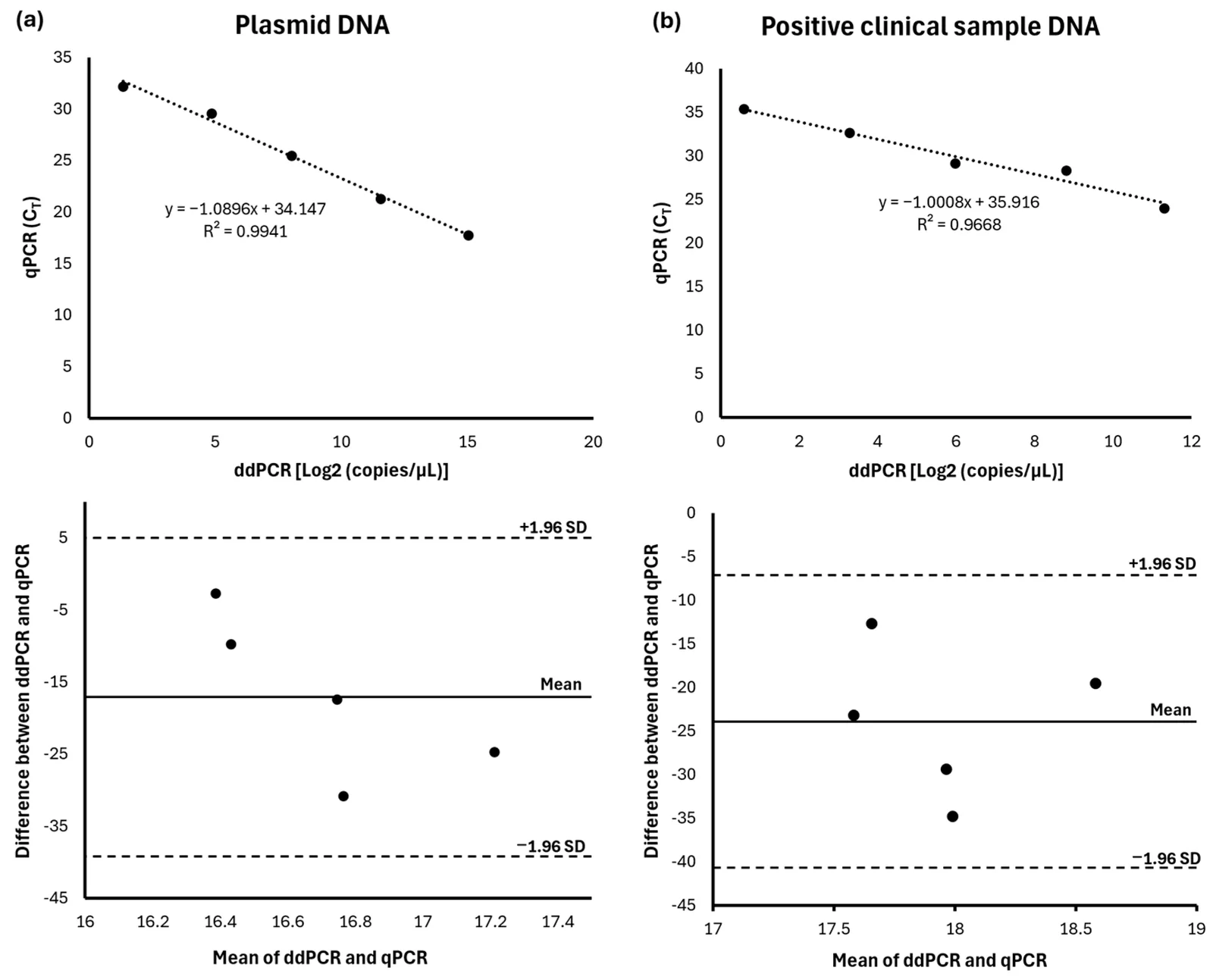
Statistical comparison between qPCR and ddPCR. (**a**) Plasmid DNA correlation between qPCR and ddPCR and the Bland–Altman plot. (**b**) Positive clinical sample DNA correlation between qPCR and ddPCR, and the Bland–Altman plot. Correlation between qPCR and ddPCR was performed using log_2_ (copies/µL) obtained by ddPCR and the number of C_T_ obtained by qPCR. The Bland–Altman plots illustrate the difference between the log_2_ (copies/µL) obtained by ddPCR and the C_T_ values obtained by qPCR for each sample, plotted against the mean of these two values. The dotted lines represent the mean ±1.96 SDs.

Linear regression analysis and the Bland–Altman method were conducted to assess methodological concordance between qPCR-derived C_T_ values and absolute target concentrations (copies/µL) determined by ddPCR (Figure 5). The corresponding Pearson correlation coefficients (r) were −0.997 for plasmid DNA and −0.983 for positive clinical sample DNA.

The intra-assay CV for ddPCR was 8.9% for ddPCR and 0.3% for qPCR, indicating good reproducibility across the evaluated concentration range for both techniques. These CV values should be interpreted within the context of the quantification method used.

### 3.5. Validation Analysis

A total of 170 blood samples from domestic animals were analysed using qPCR and the ddPCR assay developed for this study. Overall, 156 samples corresponded to canids and 14 to felids. The median age of all the animals included in the study was 6.3 years, and 63.5% were males. Six of the 170 samples (canids and felids) tested positive for *Rickettsia* spp., corresponding to a prevalence of 3.5% (Table 1, Supplementary Table S2). *Rickettsia* spp. was detected in 3.8% (6/156) of canids, whereas all felids tested negative. The infected animals included five males and one female. Their ages ranged from 4 to 11.5 years, and they all belonged to the island of Tenerife (distributed in the municipalities of El Sauzal, Tacoronte, Santa Cruz de Tenerife, and La Matanza de Acentejo).

Overall, the molecular diagnostic analysis of the samples demonstrated a high level of agreement (98.8%) between qPCR and ddPCR, resulting in two discordant samples that were negative by qPCR but positive by ddPCR. The agreement between both methods was further supported by a substantial Cohen’s kappa coefficient (κ = 0.79).

The seasonal distribution of *Rickettsia* spp. found in the canine population analysed in this study resulted in two individuals detected in winter, two in summer, one in spring, and one in autumn, which suggests a year-round pattern of infection.

## 4. Discussion

The objective of this study was to establish a rapid and efficient method for the detection of *Rickettsia* spp. in animal samples. A novel ddPCR assay was developed through the optimization of critical parameters essential for optimal PCR amplification. The resulting technique enables the specific detection of this pathogen with a limit of detection of 0.064 ng of DNA. Our results demonstrated the effectiveness of this molecular diagnostic method, allowing the validation of the technique in the field through the detection of *Rickettsia* spp. DNA in six canids from Tenerife Island.

Within the One Health approach, animal health is as important as human health, particularly in domestic animals. Canids and felids play a key role as a link between humans and the environment and remain in close contact with people, making them valuable hosts for the surveillance of zoonotic pathogens [10,11,12]. This group of bacteria is involved in both human and animal diseases; therefore, the development of optimal and robust diagnostic methods and the estimation of their prevalence in our environment are essential [2,12,13].

To our knowledge, this study represents the first description of a droplet digital PCR (ddPCR) assay for the specific detection of *Rickettsia* spp. This molecular technique was selected because it offers several advantages for pathogen detection and has been widely used in the diagnosis of a broad range of microorganisms, including bacteria, viruses, and protozoa [18,19,20,21,30,31,32,33,34,35]. Moreover, ddPCR is particularly well suited for the detection of low DNA concentrations and samples containing PCR inhibitors, characteristics commonly associated with DNA isolations obtained for *Rickettsia* detection [36,37].

The parameters evaluated in this study included annealing temperature, primer and probe concentration, and LOD. These factors are essential for accurate quantification and for the reliable detection of low-level targets in ddPCR assays, which is crucial for the proper diagnosis of infectious diseases. Furthermore, establishing the LOD is fundamental for the accurate interpretation of samples with very low bacterial loads, such as those encountered in early-stage or chronic infections, which are often underestimated in diseases such as rickettsiosis.

The ddPCR assay was developed using known copy numbers of the DNA target, as well as a positive clinical sample. Previous studies have demonstrated that this technique provides higher sensitivity and specificity than other diagnostic methods traditionally used, such as microscopy, serology, conventional PCR, and real-time PCR [18]. In addition, ddPCR offers several advantages that make it a particularly valuable diagnostic approach: no need for cultures, independence from standard curves, and the ability to achieve absolute quantification of DNA [38,39]. This method enables the specific detection of *Rickettsia* spp. at very low DNA concentrations and is less affected by PCR inhibitors, appearing to be a rapid and reliable tool for the diagnosis and monitoring of rickettsial infections in clinical samples. Nevertheless, further studies are required to fully validate the assay and assess its performance across a broader range of samples.

The specificity of the primer–probe set was evaluated in silico were plasmid DNA alignment results demonstrated high identity with several *Rickettsia* species, supporting the suitability of the selected region for subsequent detection assays. In addition, specificity was tested against the genera *Anaplasma* and *Ehrlichia*, and no amplification was observed.

A strong inverse linear correlation was observed between qPCR C_T_ values and the absolute target concentrations determined by ddPCR for both plasmid DNA (r = −0.99; R^2^ = 0.99) and positive clinical sample DNA (r = −0.98; R^2^ = 0.96). As expected, higher target concentrations measured by ddPCR were associated with lower C_T_ values in qPCR, reflecting the expected quantitative relationship between target abundance and amplification cycle thresholds. Furthermore, Bland–Altman analysis was included as a descriptive tool to visualize the relationship between qPCR C_T_ values and ddPCR-derived DNA concentrations. Given that C_T_ values and log_2_ copies/µL represent different measurement scales. These results are indicative of a strong inverse correlation between qPCR C_T_ values and the absolute DNA concentrations determined by ddPCR, consistent with the expected relationship between amplification cycles and target abundance.

However, an important methodological distinction between the techniques should be considered. Quantification by qPCR is inherently dependent on amplification efficiency and therefore relies on the performance of the specific primer-probe set and the accuracy of calibration curves. In contrast, ddPCR enables absolute quantification through sample partitioning and endpoint detection, with target concentrations calculated using Poisson statistics. As a result, ddPCR measurements are less influenced by amplification efficiency, providing improved robustness and reproducibility, particularly when detecting low-abundance targets.

Nevertheless, the developed assay presents certain disadvantages. ddPCR is highly instrument-dependent, and the cost per reaction is comparatively higher than for other molecular diagnostic methods [38,40]. Consequently, the implementation of this assay in field settings or in resource-limited regions may be challenging.

In the field validation phase of our study, *Rickettsia* spp. DNA was detected in six clinical samples during setup of the ddPCR assay. The positive cases were dogs under routine veterinary evaluation from Tenerife, Canary Islands. Although the number of positive animals was limited, these findings provide evidence of *Rickettsia* spp. exposure among domestic dogs in Tenerife and highlight the importance of incorporating sensitive molecular tools into veterinary diagnostic practice. Dogs are considered valuable sentinels for vector-borne pathogens because of their exposure to arthropod vectors and their close contact with humans, making them particularly useful for monitoring the circulation of zoonotic agents within a One Health framework [10].

The presence of several pathogenic *Rickettsia* spp. in ticks collected from dogs, birds, wildlife and humans has been previously reported in Spain. These studies reported prevalence rates of *Rickettsia* spp. detection ranging from 3.4% to 22% [41,42,43,44,45,46]. In the Canary Islands, the brown dog tick (*Rhipicephalus sanguineus* sensu lato) has been reported in association with canids and is an ectoparasite of veterinary relevance [47]. This tick species plays an important role in the epidemiology of several *Rickettsia* spp. affecting animals and humans, including *Rickettsia conorii*, the causative agent of Mediterranean spotted fever in humans, which is endemic in Spain, particularly in southern regions. Additionally, other pathogenic *Rickettsia* spp. have been identified in the country, such as *Rickettsia slovaca*, *Rickettsia raoultii*, and *Candidatus Rickettsia rioja*. In the Canary Islands, fewer studies have been conducted. Nevertheless, the presence of *Rickettsia hoogstraalii*, *Rickettsia helvetica*, and an unidentified *Rickettsia* spp. has been reported in ticks from Tenerife, showing a prevalence of 12% [48]. Fernández de Mera et al. collected and identified two tick species in Gran Canaria, *Rhipicephalus pusillus* and *Hyalomma lusitanicum*, and analyzed the presence of *Rickettsia* spp. by RT-PCR, detecting *Rickettsia massiliae* in two out of 90 ticks (2.2%) [49].

Previous research by Bolaños-Rivero et al. has reported a seroprevalence of murine typhus, caused by *R. typhi*, of 3.9% in the Canary Islands, with peaks in summer and autumn [50]. Similarly, Robaina-Bordón et al. evaluated 221 human cases of murine typhus in Gran Canaria and reported frequent contact with animals (88.7%), particularly dogs (66%), and peak incidence occurring between summer and fall [51]. Consistently, Vélez-Tobarias et al. (2025) established murine typhus as the primary etiology for non-focalized fever in La Palma and El Hierro islands [52]. Although these studies were conducted in human populations, they indicate the *Rickettsia* spp. circulation in the Canary Islands and the importance of considering the animal–vector–human interface when investigating rickettsial pathogens in this region.

Despite the limited sample size, the seasonal distribution of positive samples aligns with broader epidemiological findings for murine typhus in this region [52], which indicate a significant prevalence during the winter months. This continuous transmission throughout the year is likely supported by the archipelago’s subtropical climate, which enables the uninterrupted life cycle of flea and tick vectors. The presence of positive cases across all seasons, particularly the notable detection during winter, underlines the role of domestic pets as potential sentinels for rickettsial pathogens and highlights a persistent zoonotic risk in this subtropical environment.

This study has several limitations. Firstly, only a small number of animals tested positive for *Rickettsia* spp., likely reflecting the low occurrence of infection in the study population. Consequently, the statistical power of the study was limited. Future studies including a larger number of clinically suspected dogs and cats are needed to confirm our findings and to enable multivariable analyses accounting for potential confounding factors. Secondly, because the assay was designed for genus-level detection, positive samples were not characterized at the species level. Consequently, the pathogenic species involved could not be identified. Future studies should incorporate sequencing or species-specific molecular assays to characterize the circulating *Rickettsia* species and better assess their clinical and epidemiological significance. Thirdly, although no cross-reactivity was observed with *Anaplasma* spp. and *Ehrlichia* spp. DNA, further validation is required to comprehensively evaluate the assay’s analytical and diagnostic performance. Assessing a well-characterized panel of clinically confirmed *Rickettsia*-negative animals to determine the false-positive rate, together with a broader panel of vector-borne bacterial pathogens, including phylogenetically related organisms such as *Wolbachia* and *Orientia* and other clinically relevant bacteria affecting dogs and cats, such as *Bartonella* spp., to further evaluate potential cross-reactivity. Lastly, samples were collected retrospectively during routine clinical practice, and information regarding prior antimicrobial treatment was not consistently available for all animals. In addition, further studies evaluating the assay’s repeatability, reproducibility, and overall analytical performance are required to complete its analytical validation.

## 5. Conclusions

In conclusion, the ddPCR assay developed in this study showed promising analytical performance for the molecular detection of *Rickettsia* spp. DNA in clinical canine and feline samples. The assay has the potential to become a valuable tool for veterinary diagnostics, particularly for the detection of low-abundance *Rickettsia* DNA in clinical specimens. However, additional analytical and clinical validation studies, including assessments of assay precision, reproducibility and diagnostic performance in larger and more diverse sample sets, are required before its implementation as a routine diagnostic method. Once fully validated, this assay may also facilitate future epidemiological investigations to improve our understanding of the occurrence and distribution of *Rickettsia* spp. in animal populations and their potential public health significance.

## Figures and Tables

**Table 1:** Distribution of positive and negative samples of *Rickettsia* spp.

Canary Islands	Studied Animal: Felids, F (*n*) and Canids, C (*n*)	qPCR Detection *n* (%; CI95%)	ddPCR Detection *n* (%; CI95%)
Tenerife	C (129) F (12)	4 (2.3%; 0.9–5.9%)	6 (3.5%; 1.6–7.5%)
La Palma	C (25)	0	0

Note: Data are presented as *n* (%). Animals from Gran Canaria (1 canid and 1 felid) and Fuerteventura (1 canid and 1 felid) were also included in the study, resulting in a negative result by either qPCR or ddPCR. CI95%: Confidence intervals 95%, estimated using the Wilson Score method. The four positive samples by qPCR were also positive by the developed ddPCR method.

## Data Availability

The original contributions presented in this study are included in the article/Supplementary Materials. Further inquiries can be directed to the corresponding author.

## Supplementary Materials

The following supporting information can be downloaded at: https://www.mdpi.com/article/10.3390/vetsci13080761/s1, Table S1: Results of the *Rickettsia conorii* control DNA serial dilutions analyzed by ddPCR, and Table S2: Quantification results of the positive samples analyzed by ddPCR.
