# Supplementary material for: Development and Analytical Evaluation of a Novel Droplet Digital PCR Assay for Molecular Detection of Rickettsia spp. in Dogs and Cats from the Canary Islands
Source: Vet Sci. 2026 Jul 30;13(8):761. doi: 10.3390/vetsci13080761 (PMC13517463; doi:10.3390/vetsci13080761)
Supplement: Supplementary file 1 [file vetsci-13-00761-s001.zip › vetsci-4445147-supplementary.pdf]

Table S1. Results of the *Rickettsia conorii* control DNA serial dilutions analyzed by ddPCR

Well	Sample description	Conc. (copies/μL)	Accepted Droplets	Positives	Negatives
A08	40 ng	230.20	16617	2779	13838
B08	8 ng	40.67	18011	573	17438
C08	1.6 ng	5.73	17821	81	17740
D08	0.32 ng	0.88	18515	13	18502
E08	0.064 ng	0.25	20224	4	20220
F08	0.0128 ng	0.14	18535	2	18533
G08	2.6×10^{-3} ng	0.00	18869	0	18869
H08	C−	0.00	17383	0	17383

Note: Positive and negatives droplets resulting from the control DNA serial dilution

Table S2. Quantification results of the positive samples analyzed by ddPCR

Sample description	Conc. (copies/μL)	Accepted Droplets	Positives	Negatives
S-513	6.66	20843	110	20733
S-188	2.62	19678	41	19637
S-536	2.16	17514	30	17484
S-683	0.883	18515	13	18502
S-108	0.496	17759	7	17752
S-072	0.3	20972	5	20967

Note: Positive and negatives droplets resulting from the analyzed samples
